# An automatic fascicle tracking algorithm quantifying gastrocnemius architecture during maximal effort contractions

**DOI:** 10.7717/peerj.7120

**Published:** 2019-07-02

**Authors:** John F. Drazan, Todd J. Hullfish, Josh R. Baxter

**Affiliations:** Department of Orthopaedic Surgery, University of Pennsylvania, Philadelphia, PA, USA

**Keywords:** Ultrasound, Fascicle, Muscle mechanics, Dynamometry, Gastrocnemius, Foot and ankle, Contraction, Imaging

## Abstract

**Background:**

Ultrasound has become a commonly used imaging modality for making dynamic measurements of muscle structure during functional movements in biomechanical studies. Manual measurements of fascicle length and pennation angle are time intensive which limits the clinical utility of this approach while also limiting sample sizes in research. The purpose of this study was to develop an automatic fascicle tracking program to quantify the length and pennation angle of a muscle fascicle during maximal effort voluntary contractions and to evaluate its repeatability between days and reproducibility between different examiners.

**Methods:**

Five healthy adults performed maximal effort isometric and isokinetic contractions at 30, 120, 210, and 500 degrees per second about their ankle on an isokinetic dynamometer while their medial gastrocnemius muscle was observed using ultrasound. Individual muscle fascicles and the two aponeuroses were identified by the user in the first frame and automatically tracked by the algorithm by three observers on three separate days. Users also made manual measurements of the candidate fascicle for validation. Repeatability within examiners across days and reproducibility across examiners and days were evaluated using intra-class correlation coefficients (ICC). Agreement between manual and automatic tracking was evaluated using the coefficient of multiple correlations (CMC) and root-mean-square error. Supervised automatic tracking, where the program could be reinitialized if poor tracking was observed, was performed on all videos by one examiner to evaluate the performance of automatic tracking in a typical use case. We also compared the performance our program to a preexisting automatic tracking program.

**Results:**

We found both manual and automatic measurements of fascicle length and pennation angle to be strongly repeatable within examiners and strongly reproducible across examiners and days (ICCs > 0.74). There was greater agreement between manual and automatic measurements of fascicle length than pennation angle, however the mean CMC value was found to be strong in both cases (CMC > 0.8). Supervision of automatic tracking showed very strong agreement between manual and automatic measurements of fascicle length and pennation angle (CMC > 0.94). It also had considerably less error relative to the preexisting automatic tracking program.

**Conclusions:**

We have developed a novel automatic fascicle tracking algorithm that quantifies fascicle length and pennation angle of individual muscle fascicles during dynamic contractions during isometric and across a range of isokinetic velocities. We demonstrated that this fascicle tracking algorithm is strongly repeatable and reproducible across different examiners and different days and showed strong agreement with manual measurements, especially when tracking is supervised by the user so that tracking can be reinitialized if poor tracking quality is observed.

## Introduction

Muscle shortening dynamics during contractions in the plantar flexors govern locomotor function in athletes, the elderly, and many patient populations (Kumagai et al., 2000; Abe, Kumagai & Brechue, 2000; Suzuki, Bean & Fielding, 2001; Mulier et al., 2003; Randhawa & Wakeling, 2013). The plantar flexors, despite their relatively small size compared to the muscles of the hip and knee, play a critical role in human locomotion. These muscles behave in a variety of different ways to minimize the energy expenditure needed to complete functional activities. During the stance phase of walking, the plantar flexors act mostly isometrically to facilitate elastic energy storage and return in the Achilles tendon (Fukunaga et al., 2001). However, running requires increased rates of shortening to do the positive work necessary to accelerate the body (Lichtwark, Bougoulias & Wilson, 2007). While computational models provide critical insight into muscle-tendon dynamics in response to small changes in plantar flexor structure (Nagano et al., 2007; Baxter & Hast, 2019), coupled muscle shortening and rotation is described to have complex “variable gearing” that is dependent on both load and speed (Azizi, Brainerd & Roberts, 2008) which are not present in computational models. Therefore, experimental measurements of muscle shortening dynamics are critical for both understanding movement biomechanics of different populations while also serving to improve and validate musculoskeletal models.

Structure-function relationships can be described by quantifying both muscle shortening dynamics and joint kinetics (Maganaris, 2003; Arampatzis et al., 2005). Ultrasound imaging is a popular tool for quantifying skeletal muscle structure and shortening dynamics in human subjects during functional tasks (Franchi et al., 2018). Isokinetic dynamometry provides a unique framework for measuring joint torques generated during isolated movements while controlling for load or velocity. The combination of these two measurement techniques enable researchers to study the link between muscle structure and function that underpins musculoskeletal modeling (Reeves & Narici, 2003; Zhou et al., 2012; Blazevich et al., 2009; O’Brien et al., 2010; Randhawa, Jackman & Wakeling, 2013).

Muscle shortening dynamics in pennate muscles are quantified using ultrasound by measuring changes in length and pennation of individual fascicles in a muscle belly. Unfortunately, analyzing ultrasound images acquired during functional tasks has proven to be time intensive and technically challenging. Fascicle length and pennation have traditionally been manually digitized using custom-written computer software (Maganaris, 2001; Magnusson et al., 2001; Barber, Barrett & Lichtwark, 2011), but this approach is a time intensive process when analyzing high frame rate ultrasound data. Automatic tracking routines have been developed to make these measurements using image processing algorithms (Cronin et al., 2011; Gillett, Barrett & Lichtwark, 2013; Zhou, Chan & Zheng, 2015). Cronin et al. (2011) leveraged an optical flow algorithm to quantify fascicle length and made the “Ultratrack” analysis software freely available (Farris & Lichtwark, 2016).

This tracking framework is freely available, has an intuitive interface, and reliably measures fascicle length. Ultratrack has been a major contribution to the field of functional muscle imaging as evidenced by its popularity in the biomechanics community (Hauraix et al., 2015; Clark & Franz, 2018; Hösl et al., 2018). This approach tracks the optical flow of the entire muscle rather than individual fascicles and aponeuroses. However, as noted by Gillett, Barrett & Lichtwark (2013) non-homogeneous deformations within the region of interest (ROI) violate an underlying assumption of optical flow, which may negatively impact its performance during maximal effort contractions due to the entire muscle moving relative to the transducer. This issue can potentially be avoided by tracking smaller ROIs within the image rather than the entire muscle. This approach has been used to automatically measure muscle stretch in the plantarflexors during quiet standing (Loram, Maganaris & Lakie, 2006), though to our knowledge it has not been used to track fascicle length or pennation.

The purpose of this study was to develop and validate a new fascicle tracking paradigm that directly tracks the fascicle and aponeuroses to provide measurements of fascicle length and pennation angle and make it freely available to the biomechanics community. We used this program to automatically track an individual muscle fascicle and the aponeuroses during maximal voluntary plantar flexor contractions performed on an isokinetic dynamometer throughout a range of angular velocities for five subjects. We made manual measurements of fascicle geometry to serve as a comparison for validation. We used these data to evaluate the repeatability, reproducibility, and agreement between automatic and manual measurements of fascicle length and pennation angle across three examiners across 3 days. We assessed these parameters using correlation testing and established an a priori threshold of *r* > 0.67 to demonstrate strong agreement between comparisons. In addition, we compared the performance of this new tracking program with Ultratrack on a subset of videos from one subject.

## Materials and Methods

### Study overview

This study had two discrete activities: first, we developed a fascicle tracking algorithm that provides fascicle length and pennation angle; and second, we evaluated the performance of this tracking algorithm for quantifying muscle structure during maximal effort plantar flexor contractions. We evaluated the performance of automatic tracking compared to manual tracking during a variety of plantar flexion contractions in healthy adults. We acquired dynamometer and ultrasound data while subjects performed several isometric and isokinetic maximal-effort plantar flexion contractions. Three examiners then analyzed the ultrasound data using both the automatic tracking program and a manual tracking program three times across three different days. We compared the measurements made between days and investigators using Intra-class Correlation Coefficients (ICC) to test the intra-examiner repeatability and inter-examiner reproducibility of quantifying fascicle length and pennation angle for each approach. We tested the agreement between the automatic and manual tracking approaches by using coefficient of multiple correlations (CMC) to facilitate the comparison of our results with those of previous studies. In this study, we define repeatability as the agreement between repeated measurements of the same data using identical methods. We define reproducibility as the agreement of repeated measures of the same data made by different observers and/or different methods (Bartlett & Frost, 2008). In addition, we had one examiner perform “supervised tracking” where the tracking protocol could be reinitiated if poor automatic tracking was observed. Finally, we compared the performance of our automatic tracking algorithm with the Ultratrack software within a subset of videos from a single subject.

### Fascicle tracking algorithm

We developed a custom software tool to measure fascicle length and pennation angle during plantarflexor contractions (Supplemental Material). As muscle fascicles and the aponeuroses are clearly visible using ultrasound, we developed a program using a commercially available point tracking tool to directly track these structures through a contraction. This tool uses the Kanade-Lucas-Tomasi (KLT) feature tracking algorithm for point tracking implemented within the MATLAB and the Computer Vision Toolbox (Mathworks, Natick, MA, USA) (https://www.mathworks.com/help/vision/ref/vision.pointtracker-system-object.html). We used these tracked structures to calculate fascicle length and pennation angle from their relative positions and orientations. The workflow of this tool can be broken into two phases: (1) identifying superficial and deep aponeuroses and a candidate fascicle in the first frame and (2) automatic tracking performed by the algorithm through the rest of the ultrasound video.

Identification required a user to identify the deep and superficial aponeuroses of the muscle belly as well as a single muscle fascicle in the first frame of a contraction video. The user did this by drawing a line over each of these structures. After the user confirmed that these drawn lines correctly identified the structures of interest, the automatic tracking phase began.

The following steps took place for each of the three structures identified by the user:

1. A rectangular ROI was defined along the long axis of each structure. The height and width of the ROI relative to the original structure was defined as a user input. For example, the fascicle ROI was defined as only the middle 90% of the identified fascicle in order to prevent the seeding of either aponeurosis.
2. For the deep aponeurosis, the color scale of the ROI was rebalanced because we observed that this improved tracking performance during program development.
3. Kanade-Lucas-Tomasi point tracking performs best with features composed of textured ROIs (Jianbo & Tomasi, 1993). Each structure was seeded with a user defined number of points (in this study, 100) that were evenly distributed through the ROI. Ideal tracking points were automatically identified using the detectMinEigenFeatures function in the MATLAB computer vision toolbox.
4. The MATLAB pointTracker function was used to iteratively track the position of the seeded points through the video. Fundamentally, this function uses a KLT algorithm to calculate the optical flow of a region to determine the movement of an object between two frames (Tomasi & Kanade, 1991; Lucas & Kanade, 1981). The MATLAB pointTracker function allows the user to control tracking by defining the size of the region surrounding each seeded point (block size), the number of levels contained within the image pyramid that enables multi-resolution tracking, the maximal bidirectional error, and the number of iterations over which minimization takes place. In our implantation of this function, we used a block size of three mm, a four level pyramid, a bidirectional error of two mm, and capped the number of iterations at 30. Generally, more iterations, larger block sizes, and more pyramid levels increase computational time. Decreasing the bidirectional error threshold ensures tracking quality at the cost of tracked point attrition.
5. The pointTracking program produced the coordinates of each point in the current frame, as well as logical values that defined the validity of the tracking for each point. Point validity is defined through “forward-backward” tracking where the KLT is used to track the point from the current frame back to the previous frame (Kalal, Mikolajczyk & Matas, 2010). If the distance between the backtracked point and the real point in the previous frame was greater than the bidirectional error, (in our case two mm) the point was considered invalid. In addition, if the point moved out of the frame of the video it was considered invalid. Invalid points were discarded from the point cloud. In the event that more than 10% of the points in the prior frame were invalid in the current frame, 100 new points were identified in the current frame and tracking continued. Note: If 100% of the points were dropped the program terminates and displays an error. This did not occur in any of the tracking sessions performed on our 75 videos.
6. Following the identification of the tracked points in the new frame, this point cloud was fit with linear regression to determine the relative orientation and position of the three structures.

Fascicle length and pennation angle was calculated using these structures. The intersections of the fascicle line and the aponeurotic lines were defined to be the attachment point of the tracked fascicle with the aponeuroses. Fascicle length was defined as the point to point distance between the intersection of the fascicle line with the superficial and deep aponeuroses. Pennation angle was calculated as the angle between the fascicle and the deep aponeurosis. In the event that either fascicle attachment was out of frame of the video, the lines were extrapolated to make the measurements. The software visualized the tracked point clouds and the best fit lines overlaid on the ultrasound video. Following completion of tracking, the program allowed users to accept the automatic tracking results, reprocess the trial, or validate the automatic tracking results by manually identifying the same fascicle throughout a user-defined set of frames.

In this study, we performed both automatic and manual tracking. Automatic tracking could be performed in two ways: (1) Unsupervised, where the examiner identified the fascicles and aponeuroses in the first frame and did not watch the tracked video. (2) Supervised, where the examiner watched the resulting tracked video. If the tracked fascicle was observed to not align with fascicles in the ultrasound video, the examiner could re-identify the structures in the first frame and retrack the fascicle. Following completion of automatic tracking, the user had the ability to validate the measurements by manually identifying the fascicle in a series of frames. To manually track a fascicle, the examiner drew a line over the aponeuroses and the candidate fascicle. Manual measurements of fascicle length and pennation angle were collected for isokinetic contractions at −20, −10, 0, 10, 20, and 30 degrees of ankle plantar flexion to serve as a comparison for the automatic tracking. During isometric trials, frames for manual measurements were collected at evenly spaced time indices corresponding to 0%, 20%, 40%, 60%, 80%, and 100% of the contraction. While automatic tracking provided digitized measurements for every frame, in this study, automatic tracking was down sampled to the six frame indices corresponding to the manual measurements.

### Data acquisition

Five healthy adults participated and provided written-informed consent in this study approved by the University of Pennsylvania IRB (828374). Subjects were positioned prone on a treatment table rigidly attached to a multi-mode dynamometer (System 4; Biodex, Shirley, NY, USA). The subject’s right foot was secured to a foot plate with the medial malleolus of the ankle aligned with the dynamometer’s spindle. An ultrasound transducer was affixed to the lower leg over the mid-belly of the medial gastrocnemius using a custom-made cast. Ultrasounds frames were collected with a six cm transducer (LV7.5/60/128Z-2, SmartUs, TELEMED) at a rate of 60 frames/s. Dynamometer and ultrasound data were acquired simultaneously while subjects performed isometric and isokinetic maximal effort contractions at 30, 120, 210, and 500 degrees per second. This wide range of velocities for tracking validation was selected because previous studies have demonstrated the utility of automatic fascicle tracking, developed by Cronin et al., at plantar flexion velocities ranging from 30 °/s to 701 °/s (Hauraix et al., 2015). Isometric testing was performed with the ankle in neutral (90° angle between the foot and the shank). Subjects were instructed to “push as hard and as fast as possible” for all contractions. We provided subjects verbal encouragement and visual feedback to help maximize effort during each contraction. Subjects were asked to perform multiple maximal effort contractions during each velocity condition until they produced similar peak torques for three consecutive contractions, which typically took three to five trials. We analyzed the final three trials in this study per condition per subject.

### Evaluation of repeatability, reproducibility, and agreement between manual and automatic tracking

Three examiners tracked the ultrasound data for the five subjects on three separate days to test intra-examiner repeatability and inter-examiner reproducibility for both manual and automatic approaches. For this portion of the study, automatic tracking was performed unsupervised where the first attempt at tracking was accepted. Each examiner was trained on how to identify fascicles and how to use the program on a sample ultrasound video. Each examiner independently performed both manual and automatic tracking during each session to provide a comparison between the two methods. To prevent bias, examiners were blinded to the identifying data for each trial within a subject and were explicitly instructed not to watch the automatically tracked video prior to manual tracking. Manual tracking took place following the automatic tracking of a given video. Each examiner was provided with a visual marker indicating the location of the deep insertion of the automatically tracked fascicle so that the same candidate fascicle was identified in each frame. Automatic measurements of fascicle length and pennation angle were extracted at the same frame indices (the same frame within the video) corresponding to the six manual measurements for comparison between the two methods. Three videos of contractions were analyzed for each of the five contractile conditions, across five subjects, which resulted in a total of 75 videos under analysis. Each of the three examiners tracked each video on three separate days which resulted in 675 tracked videos. Manual tracking was an intensive process and mistakes in manual tracking occurred very rarely due to errant mouse clicks. Eight instances (out of 4,050 individual manual fascicle measurements) of extreme user error in manual identification (difference between manual and automatic measurements of fascicle length greater than 40 mm) were identified and removed from analysis.

Similar studies have observed a persistent offset between repeated automatic measurements of fascicle length which reflect differences in user identified fascicles in the first frame (Cronin et al., 2011; Gillett, Barrett & Lichtwark, 2013). We corrected for this offset by subtracting the offset from each measurement so that measurements shared the same initial value. As such, reproducibility of automatic measurements of fascicle length and pennation angle were reported for both uncorrected and corrected CMC values.

We performed several correlation analyses to quantify the repeatability within examiners between days and reproducibility across examiners. The intra-examiner repeatability between days and inter-examiner reproducibility across days was calculated for both manual and automatic measurements using ICC’s in a two way mixed effects model (McGraw & Wong, 1996). We tested the absolute agreement between individual measurements (A-1 formulation) of fascicle length and pennation angle. To test intra-examiner repeatability, ICCs were calculated within examiners across days. To test inter-examiner reproducibility, ICCs were calculated across examiners and across days. Root mean square error (RMSE) was also calculated to provide a measurement of absolute agreement between methods. We also tested the reproducibility of automatic tracking by calculating the CMC values for each trial across examiners and days for both uncorrected and corrected values of fascicle length and pennation angle.

To test the agreement between manual and automatic tracking, we calculated the CMC and RMSE value for each individual trial comparing manual to automatic tracking for measurements of fascicle length and pennation angle (Kadaba et al., 1989; Queen, Gross & Liu, 2006). Both ICC and CMC tests produce *r* values ranging between 0 and 1 where higher values represented greater agreement between measurement methods. Specifically, *r* values between 0 to 0.36, 0.36 to 0.67, 0.67 to 0.9, and 0.9 to 1.0 represent poor, moderate, strong, and very strong correlations, respectively (Taylor, 1990).

### Supervised tracking in a simulated use case

**Figure 1:**
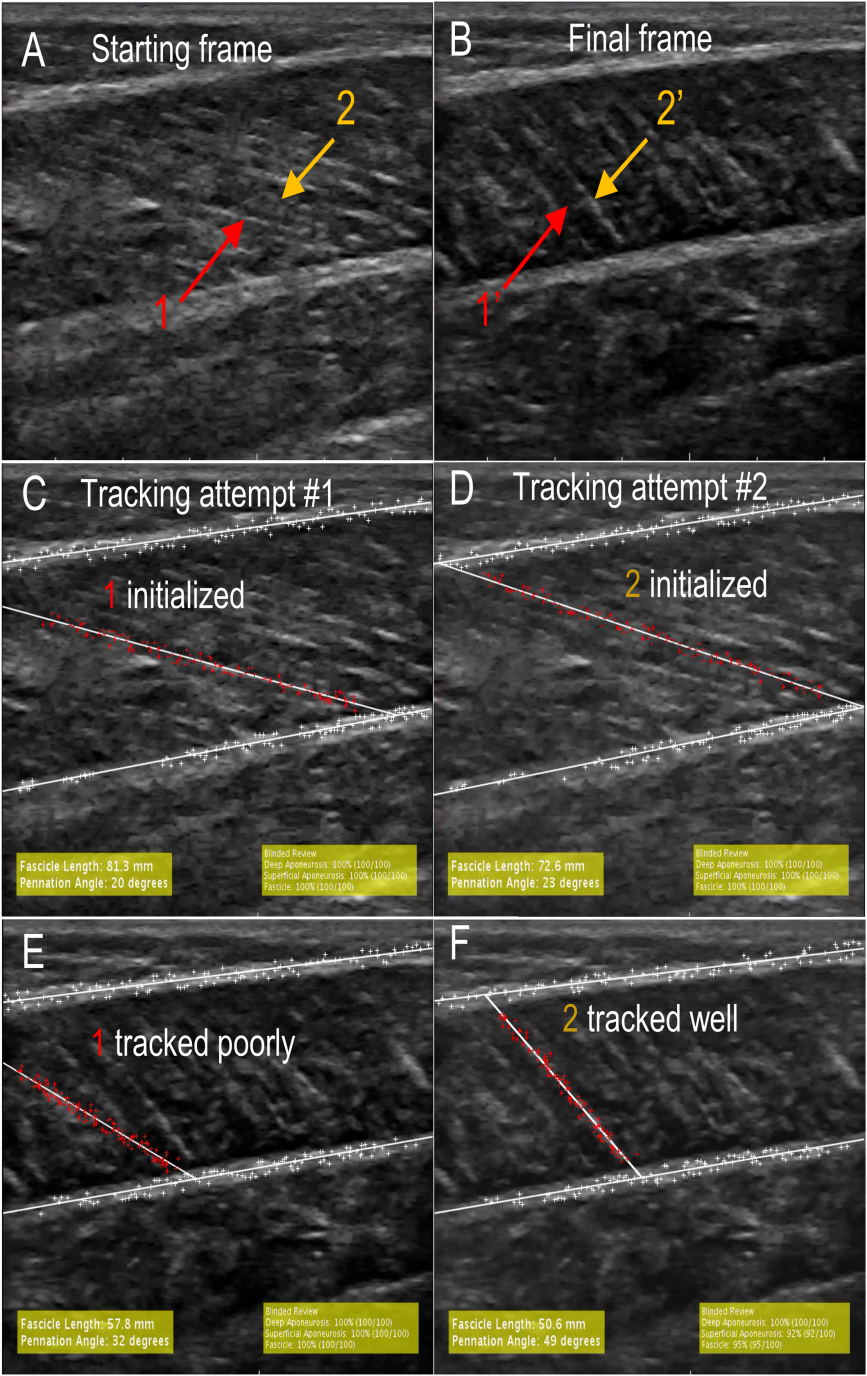
A visualization of supervised tracking. (A) During tracking initialization, the examiner selected a highly visible fascicle in the first frame of the trial (red 1) out of many candidate fascicles (orange 2). (B) When tracking is completed, the selected fascicle (red 1′) is no longer visible in the frame while a different candidate fascicle (orange 2′) is still visible. (C) Initial seeded fascicle (red 1) that disappeared during the contraction. (D) Initial seeded fascicle based on fascicle (orange 2) that remained visible during contraction. (E) Final frame of tracking based on first fascicle (red 1). In supervised tracking, this would be considered a poor track. (F) Final frame of tracking based on second fascicle (orange 2). In supervised tracked, this would be considered a good track.

While the unsupervised tracking approach is useful to test the repeatability and reproducibility of tracking across examiners, it is a poor analog for how automatic fascicle tracking is used in practice where users supervise the tracking. For example, Ultratrack includes functionality for the user to view videos prior to fascicle identification and make manual corrections (Farris & Lichtwark, 2016). While our program does not include any of these features, we sought to test the performance of our approach in a case study where users had the ability to re-initialize the program after watching a first attempt at tracking. As such, one examiner performed “supervised tracking” where the program was used to track all data from five subjects (75 videos) with the ability to re-initialize the tracking if poor tracking was observed. Poor tracking was defined as an obvious difference between the tracked fascicle and the fascicles in the ultrasound images upon review (Fig. 1E). Aeles et al. (2017) showed that watching ultrasound videos of fascicle shortening increased the reliability of manual fascicle identification relative to static images. During supervision, the examiner watched the tracked trial following program initialization, which provided information regarding fascicle geometry that was not apparent in the static first frame as well as allowed the examiner to evaluate the quality of the tracking. Upon observing a poorly tracked trial, the examiner rejected the tracking, re-identified the aponeuroses, and identified the same or different candidate fascicle based on watching the video (Fig. 1).

Agreement between automatic and manual tracking using the supervised approach was evaluated using CMCs and RMSEs. The Bland–Altman method of differences was used to evaluate the agreement between the two approaches (Bland & Altman, 1986). Bland–Altman analysis does not have a defined threshold for statistical acceptance and instead relies on establishing an acceptable threshold a priori based on application specific requirements (Giavarina, 2015). A recent review reported coefficient of variation ranging from 0% to 9.8% for measurements of either fascicle length and pennation angle in the human gastrocnemius across a range of measurement approaches (Kwah et al., 2013). As such, we established an a priori coefficient of variation value of 10% for both fascicle length and pennation angle.

To quantify whether errors increased over time due to the accumulation of errors due to tracking, we performed an analysis of the isokinetic data (30, 120, 210, and 500 °/s) where we calculated the RMSE between all manual and automatic measurements of fascicle length and pennation angle at each of the six ankle positions. We calculated this for both unsupervised and supervised tracking approaches.

### Comparison with existing fascicle tracking tool

One examiner compared the performance of our tracking algorithm to the Ultratrack 4.2 software that was downloaded from https://sites.google.com/site/ultratracksoftware/home on five videos, one for each contraction condition from one subject. This tracking was performed “unsupervised,” where the first track was accepted for all tracking approaches regardless of quality. Prior to this experiment, the examiner practiced with Ultratrack using sample videos outside of the subset to gain familiarity with the software. The software was used based on information found in Farris & Lichtwark (2016) in accordance with instructions on the website. As noted by previous users of this tool (Clark & Franz, 2018), Ultratrack does not provide a true measurement of pennation angle when used in the manner as described in Farris & Lichtwark (2016) Rather, it provides the angle between the identified fascicle and the horizontal axis of the video.

First, we used Ultratrack in the same manner as we used our own tracking approach where fascicle and aponeuroses were identified in the first frame. We identified a ROI that contained the aponeuroses and the entire muscle belly and allowed the ROI to change during the contraction by deselecting the “Fixed ROI” button. Then, we identified an individual fascicle where the deep attachment was visible in the frame, initialized tracking, and exported the resulting ROI geometry, fascicle length, and pennation angle data for further analysis. Second, we used the ROI and fascicle position data that was used to initialize the Ultratrack tracking as inputs into our own tracking program and performed tracking using identical starting conditions. During preliminary testing we noticed that Ultratrack performed poorly when one or both of the fascicle attachments were out of frame. Ultratrack has the capability to identify a fascicle and ROI within a middle frame, track forward to the end frame and then track backward to the first frame. Ultratrack performs best when the candidate fascicle is identified with both attachments in the frame. Therefore, to provide another comparison, we also performed tracking with Ultratrack that was initialized in a later frame where the entire fascicle was visible. Six evenly spaced manual measurements of fascicle length and pennation angle were collected for each condition for comparison to these three automatic measurement approaches. All data is represented as mean ± standard deviation unless otherwise noted.

## Results

Automatic and manual measurements of fascicle length and pennation angle across all isometric and isokinetic conditions were found to be repeatable with strong intra-examiner agreement (ICC > 0.74, Table 1). These measurements were also found to be reproducible with strong inter-examiner agreement across all examiners and days (ICC > 0.76, Table 1). Automatic measurement of fascicle length and pennation had larger RMSE values relative to manual measurements (Table 1). Fascicle length measurements were more reproducible than measurements of pennation angle. The automatic measurements were less reproducible than the manual measurements, however, automatic tracking reproducibility and repeatability remained strong for all comparisons.

**Table 1:** ICC comparisons and RMSE for intra-examiner repeatability and inter-examiner reproducibility.

		Intra-observer		Inter-observer	
		Automatic	Manual	Automatic	Manual
Fascicle length ICC (RMSE (mm))	Examiner 1	0.92 (4.21)	0.94 (4.01)	0.86 (5.09)	0.93 (3.87)
	Examiner 2	0.91 (3.93)	0.95 (3.29)		
	Examiner 3	0.84 (5.85)	0.93 (3.87)		
Pennation angle ICC (RMSE (°))	Examiner 1	0.86 (3.33)	0.94 (2.92)	0.76 (4.61)	0.90 (3.43)
	Examiner 2	0.84 (4.07)	0.91 (3.48)		
	Examiner 3	0.74 (4.83)	0.92 (2.89)		

**Figure 2:**
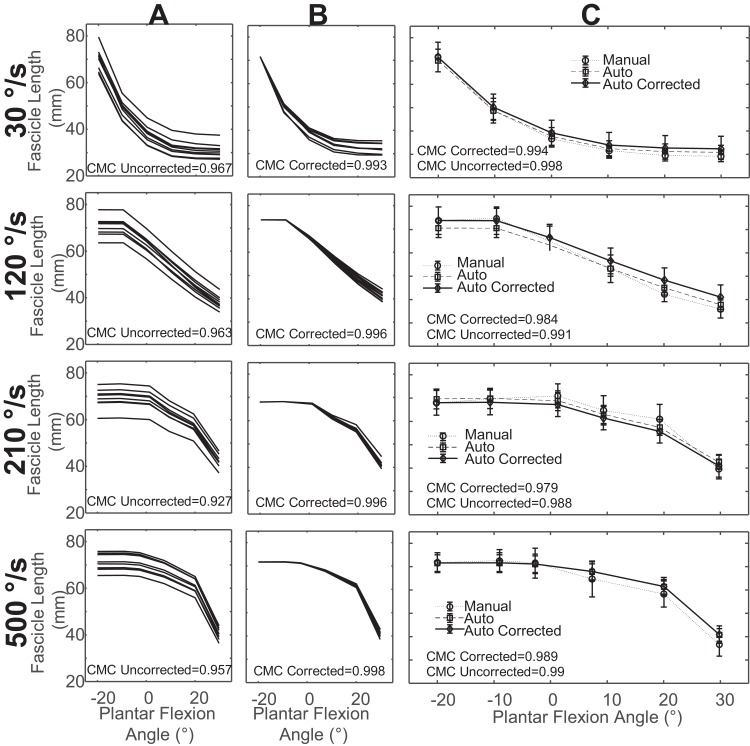
Sample data for fascicle length measurements from a single subject. (A) overlay of fascicle length measurements in one trial for all examiners across all days prior to correction for initial offset. (B) Overlay of fascicle length measurements in one trial for all examiners across all days after correction for initial offset. (C) Mean fascicle length measurements across all examiners and days for uncorrected, corrected, and manual measurements with standard deviation.

**Figure 3:**
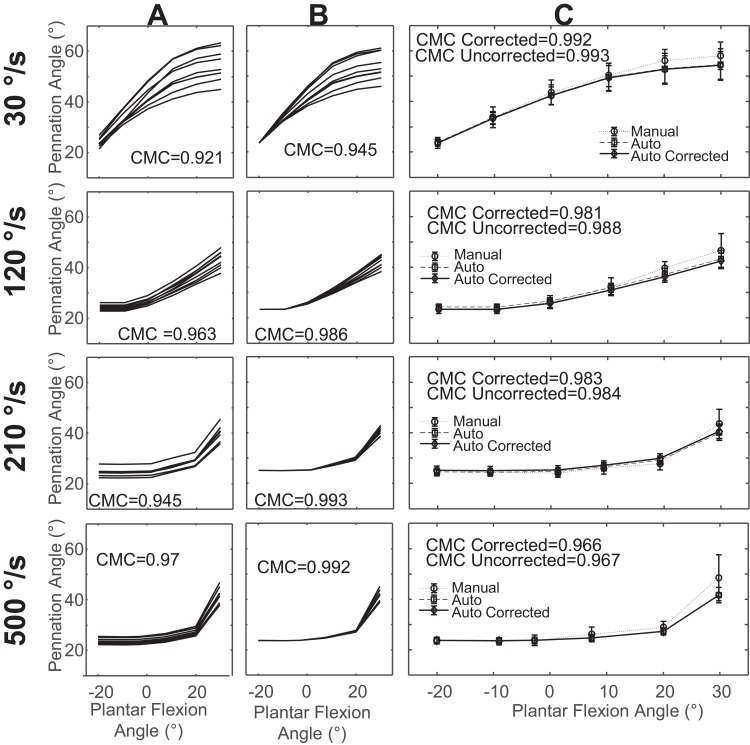
Sample data of pennation angle measurements from a single subject. (A) Overlay of pennation angle measurements in one trial for all examiners across all days prior to correction for initial offset. (B) Overlay of pennation angle measurements in one trial for all examiners across all days after correction for initial offset. (C) Mean pennation angle measurements across all examiners and days for uncorrected, corrected, and manual measurements with standard deviation.

We found that the reproducibility of automatic tracking was strong for uncorrected measurements and very strong in corrected measurements of fascicle length (Figs. 2A and 2B) and pennation angle measurements (Figs. 3A and 3B). Corrected values for fascicle measurements had a higher mean CMC value with a smaller standard deviation (CMC = 0.98 ± 0.02) relative to the uncorrected values (CMC = 0.88 ± 0.09). Automatic tracking reproducibility of pennation angle was also improved following initial bias correction (CMC = 0.92 ± 0.04) compared to uncorrected values (CMC = 0.84 ± 0.1).

Agreement between automatic and manual measurements of fascicle length was very strong in both uncorrected (CMC = 0.90 ± 0.13) and corrected (CMC = 0.93 ± 0.10) values (Table 2). Isometric conditions resulted in the higher CMC values and smaller errors relative to the isokinetic conditions (Table 2). There was greater error in the uncorrected values (RMSE = 5.86 ± 4.13 mm) relative to the corrected values (RMSE = 4.97 ± 3.65 mm) (Table 2). The RMSE was highest at 500 °/s and lowest for isometric, although isometric had the highest RMSE standard deviation.

**Table 2:** CMC and RMSE calculations to test agreement between manual and unsupervised automatic tracking measurements of fascicle length.

Condition	Mean CMC (standard deviation)		Mean RMSE (standard deviation) (mm)	
	Length	Length corrected	Length	Length corrected
30 °/s	0.87 (0.19)	0.91 (0.13)	5.75 (4.41)	4.24 (3.56)
120 °/s	0.93 (0.11)	0.93 (0.08)	5.7 (4.08)	5.23 (3.29)
210 °/s	0.9 (0.13)	0.92 (0.12)	5.72 (3.37)	5.21 (3.75)
500 °/s	0.88 (0.13)	0.91 (0.13)	6.61 (3.74)	5.63 (3.94)
Isometric	0.94 (0.09)	0.96 (0.05)	5.54 (6.63)	4.55 (3.69)
Mean	0.90 (0.13)	0.93 (0.10)	5.86 (4.13)	4.97 (3.65)

Agreement between automatic and manual measurements of pennation angle was strong in both uncorrected (CMC = 0.83 ± 0.18) and corrected (CMC = 0.86 ± 0.15) values (Table 3). Isometric conditions resulted in the higher CMC values and smaller errors relative to most of the isokinetic conditions (Table 3). There was greater error in the uncorrected values (RMSE = 7.16° ± 4.25°) relative to the corrected values (RMSE = 6.22° ± 3.90°) (Table 3). The RMSE was lowest for isometric for measurements of pennation angle in contrast to fascicle length in which it was highest.

**Table 3:** CMC and RMSE calculations to test agreement between manual and unsupervised automatic tracking measurements of pennation angle.

Condition	Mean CMC (standard deviation)		Mean RMSE (standard deviation) (°)	
	Pennation	Pennation corrected	Pennation	Pennation corrected
30 °/s	0.78 (0.24)	0.83 (0.17)	8.04 (4.77)	6.47 (4.22)
120 °/s	0.85 (0.17)	0.85 (0.16)	7.32 (4.46)	6.84 (4.07)
210 °/s	0.84 (0.16)	0.87 (0.15)	6.7 (3.76)	6 (3.46)
500 °/s	0.81 (0.17)	0.87 (0.13)	7.75 (4.43)	6.35 (4.03)
Isometric	0.85 (0.16)	0.87 (0.14)	5.99 (3.85)	5.46 (3.72)
Mean	0.83 (0.18)	0.86 (0.15)	7.16 (4.25)	6.22 (3.9)

**Figure 4:**
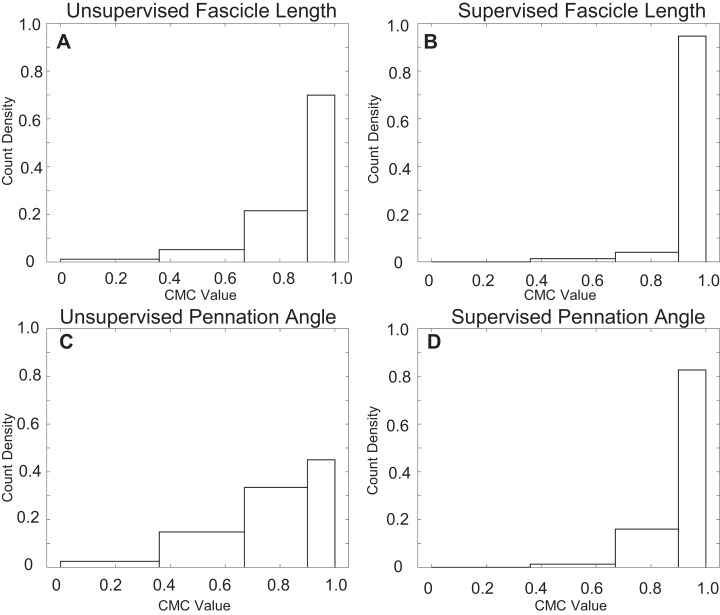
Count density histograms for all CMC values comparing manual to automatic tracking. Distributions of CMC values comparing manual and automatic measurements. Values were grouped by poor, moderate, strong, and very strong from left to right. (A) Distribution of CMC values for uncorrected fascicle length measurements. (B) Distribution of CMC values for corrected fascicle length measurements. (C) Distribution of CMC values for uncorrected pennation angle measurements. (D) Distribution of CMC values for corrected pennation angle measurements.

Supervised automatic tracking demonstrated very strong agreement between manual and automatic measurements of both fascicle length (CMC = 0.97 ± 0.04) and pennation angle (CMC = 0.94 ± 0.06) as well as a considerably smaller RMSE relative to unsupervised (Table 4). Supervision sharply reduced the incidence of tracking trials with poor and moderate agreement with over 90% of fascicle length measurements and over 80% of pennation angle measurements having very strong agreement between manual and automatic approaches (Fig. 4).

**Table 4:** CMC and RMSE calculations to test agreement between manual and supervised automatic tracking measurements for fascicle length and pennation angle.

Condition	Mean CMC (standard deviation)		Mean RMSE (standard deviation)	
	Length	Pennation	Length (mm)	Pennation (°)
30 °/s	0.96 (0.09)	0.93 (0.11)	2.5 (1.39)	3.83 (1.7)
120 °/s	0.98 (0.01)	0.96 (0.05)	3.24 (1.26)	3.62 (2.05)
210 °/s	0.97 (0.05)	0.95 (0.05)	3.09 (1.76)	4.23 (2.15)
500 °/s	0.95 (0.04)	0.93 (0.04)	4.42 (1.43)	4.84 (2.00)
Isometric	0.98 (0.01)	0.94 (0.05)	3.31 (1.79)	4.09 (1.99)
Mean	0.968 (0.04)	0.942 (0.06)	3.312 (1.53)	4.122 (1.98)

**Figure 5:**
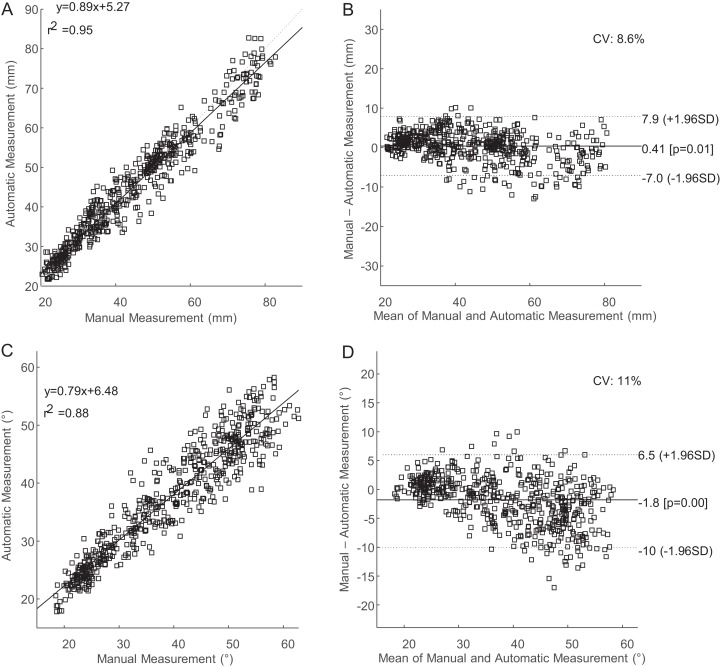
Regression and Bland–Altman plots comparing supervised automatic tracking to manual tracking. (A) Regression plot comparing manual to automatic measurements of fascicle length. (B) Bland–Altman plot comparing manual to automatic measurements of fascicle length. (C) Regression plot comparing manual to automatic measurements of pennation angle. (D) Bland–Altman plot comparing manual to automatic measurements of pennation angle.

The coefficient of variation for supervised measurement of fascicle length and pennation angle was 8.6% and 11%, respectively (Fig. 5). The coefficient of variation for pennation angle fell slightly outside of our a priori threshold of 10% while fascicle length met this criterion. Despite the strong agreement between the automatic and manual tracking approaches, the unsupervised automatic tracking under-approximated fascicle lengths by 5.6% and over-approximated pennation angles by 10.1% across the entire range of motion. Supervision of automatic tracking decreased these errors to less than 1.1% for under-approximation of fascicle length and 5% over-approximation for pennation angle. The values are mean signed errors.

With regards to quantifying the accumulation of error over the course of a video, we observed that RMSE in fascicle length decreased over time for both unsupervised and supervised tracking. In contrast, RMSE in pennation angle increased over time for unsupervised and supervised tracking. In both cases, supervision approximately halved the RMSE error, especially in late frames (Table 5).

**Table 5:** RMSE calculation for fascicle length and pennation angle during isokinetic contractions as a function of ankle angle.

		RMSE through isokinetic contraction (°plantar flexion)					
		−20°	−10°	0°	10°	20°	30°
Fascicle length(mm)	Unsupervised	7.81	7.49	7.4	7.07	6.64	6.59
	Supervised	4.78	3.7	3.77	3.41	3.28	2.9
Pennation angle (°)	Unsupervised	3.2	5.83	8.06	9.66	10.94	11.29
	Supervised	1.99	2.78	4.13	5.13	5.66	6.3

**Figure 6:**
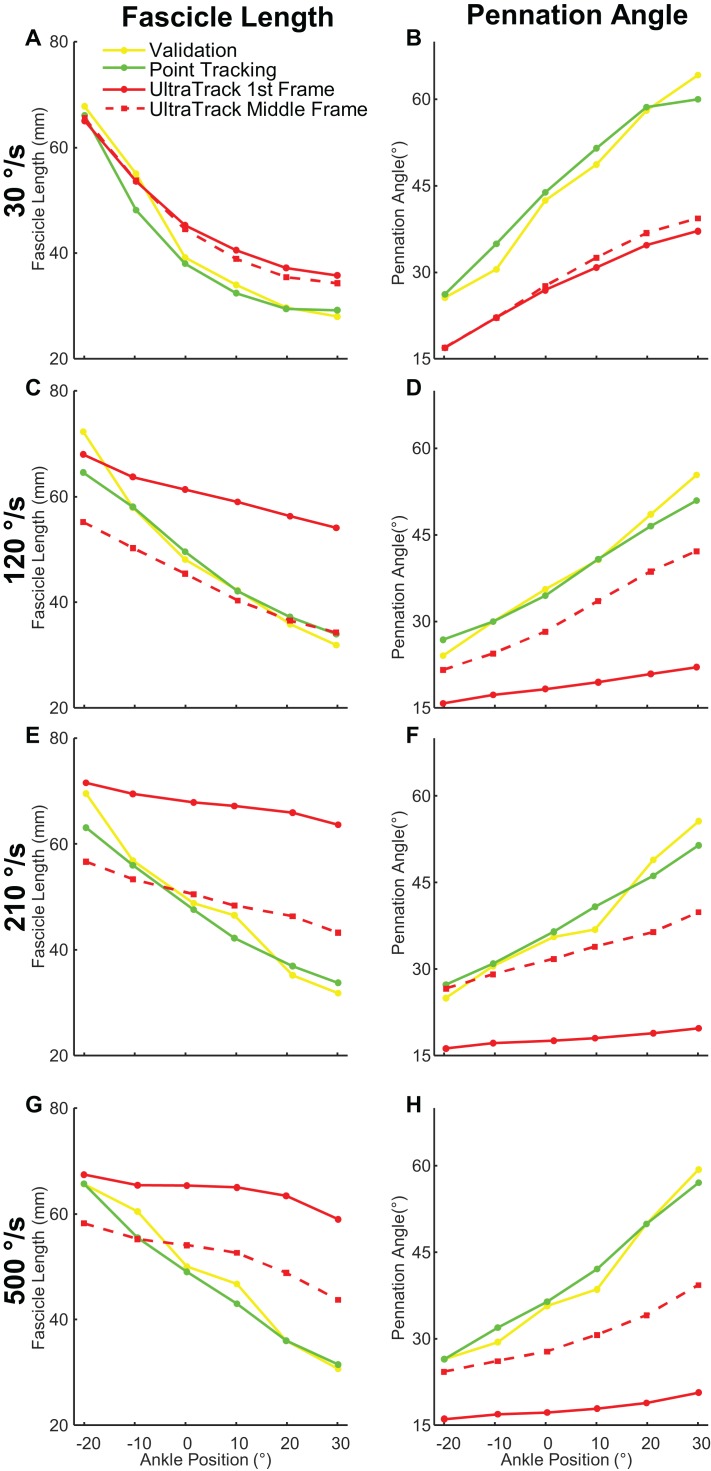
Comparison of manual, point tracking, and Ultratrack measurements of fascicle length and pennation angle. Yellow shows manual measurements. Green is our point tracking approach. Solid red is the Ultratrack initialized from the first frame. Red dashed is Ultratrack initialized from a frame drawn from the middle of a contraction where the fascicle is fully in view. (A) Fascicle length measurements at 30 °/s, (B) pennation angle measurements at 30 °/s, (C) fascicle length measurements at 120 °/s, (D) pennation angle measurements at 120 °/s, (E) fascicle length measurements at 210 °/s, (F) pennation angle measurements at 210 °/s, (G) fascicle length measurements at 500 °/s, (H) pennation angle measurements at 500 °/s.

**Figure 7:**
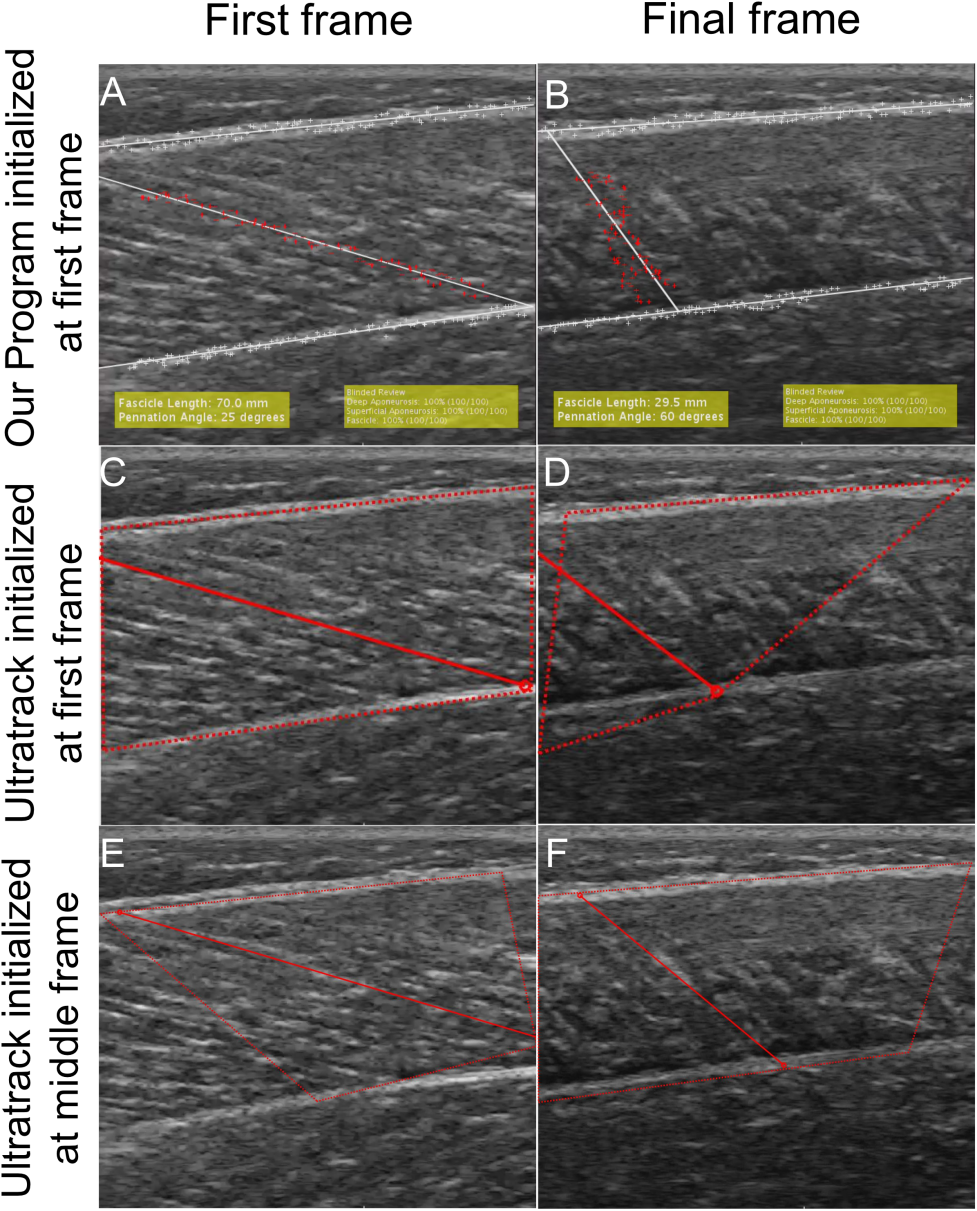
Visualization of tracking between our program and Ultratrack initialized from the first frame and middle frame. To evaluate the performance of our tracking program and Ultratrack, we tracked a subset of videos with both programs. (A) First frame of Ultratrack where the fascicle and ROI was defined in the first frame. (B) The final frame of Ultratrack with a tracked fascicle where the fascicle and ROI was defined in the first frame. (C) The first frame of our program with the same starting information as Ultratrack in (A). (D) The final frame of our program with a tracked fascicle. (E) The first frame of Ultratrack when the candidate fascicle and ROI was defined in a middle frame when the entire fascicle is in view. (F) The final frame of Ultrack initialized from a middle frame.

Our program produced smaller tracking errors compared to Ultratrack when provided with the same starting fascicle and aponeuroses geometry for measurements of both fascicle length and pennation angle (Fig. 6). Initializing Ultratrack from a middle frame improved tracking fidelity but these errors were still greater than our approach (Fig. 7). Initializing in the middle frame reduced errors in Ultratrack in all cases outside of isometric max (Table 6).

**Table 6:** RMSE comparison between our point tracker program and Ultratrack.

		30 °/s	120 °/s	210 °/s	500 °/s	Isometric	Mean
This work	RMSE of fascicle length (mm)	3.05	3.35	3.38	2.61	4.74	3.43
Ultratrack 1st frame		5.88	15.35	22.03	18.92	9.42	14.32
Ultratrack middle frame		4.71	7.83	8.56	8.84	11.33	8.25
This work	RMSE of pennation angle (°)	2.81	2.33	2.8	2.04	2.88	2.18
Ultratrack 1st frame		18.19	21.79	22.79	24.12	18.26	22.96
Ultratrack middle frame		16.83	8.32	8.5	11.51	11.62	9.92

## Discussion

The purpose of this study was to establish the reproducibility, repeatability, and agreement of a novel automatic fascicle tracking algorithm that directly tracks the fascicle and aponeuroses. This algorithm was tested with five subjects during maximal voluntary contractions performed on an isokinetic dynamometer in isometric and isokinetic conditions. Our results indicate that this automatic tracking approach is repeatable, reproducible, and had strong agreement with manual measurements for three different examiners across three different days. We showed that supervision of the automatic tracking and reinitializing the program as needed provided accurate measurements of both fascicle length and pennation angle in one tracking session. Also, we showed that this tracking approach exhibited a smaller RMSE than the popular Ultratrack software when tracking the same fascicle. Our tool also provides the user with pennation angle measurements. The reliability of the automatic tracking coupled with its speed relative to manual tracking makes this approach an attractive method for studying muscle geometry during maximal effort contractions.

The agreement between automatic and manual fascicle length measurements compared well with the literature. Our repeatability measurements for manual measurements of fascicle length and pennation angle fall within previously reported values (0.87–0.99 for fascicle length and 0.8 for pennation) for measuring muscle architecture parameters in identical images (Kwah et al., 2013). The reproducibility of automatic measurements of fascicle length (0.86) and pennation angle (0.76) across all examiners and days fell just short of these values, but remained classified as strong. Previous groups have reported mean uncorrected CMC values for fascicle length measurements of 0.88 and 0.9 which compares well with our value of 0.89 (Cronin et al., 2011; Gillett, Barrett & Lichtwark, 2013). We observed that measurements of pennation angle had less agreement than measurements of fascicle length which has also been observed by previous groups (Aggeloussis et al., 2010; Kwah et al., 2013). The repeatability of automatic fascicle length measurements for uncorrected and corrected values was 0.88 ± 0.09 and 0.98 ± 0.02, respectively. This compares well with previously reported values for Ultratrack, 0.88 ± 0.08 and 0.98 ± 0.02 for uncorrected and corrected values, respectively, (Cronin et al., 2011). While there are fewer examples of automatic pennation angle measurement in the medial gastrocnemius, one group (Zhou, Chan & Zheng, 2015) reported an average correlation of *r* = 0.935 which compares well with our value of *r* = 0.94 for supervised pennation measurement. While the coefficient of variation for pennation measurements fell slightly outside of our a priori value of 10%, an 11% coefficient of variation still provides researchers with a useful tool for the automatic tracking of fascicles.

We found that the automatic tracking was reproducible across days and examiners as demonstrated by strong agreement (ICC values > 0.76). While this value was lower than the reproducibility of our manual measurements (>0.9), it represents the worst case scenario as the program was unsupervised. While ICCs were not calculated for the supervised algorithm because only one examiner performed this testing, the high correlation between manual and automatic measurements (CMC > 0.94) and reduced RMSE provides evidence supervising automatic tracking will enhance reproducibility. Automatic tracking is significantly faster than manual tracking. To initialize the program, a user only needs to draw three lines in the first frame which takes less than 15 s. To process all the frames in a 150 frame video, it takes approximately 15 s. Manual tracking, even when only digitizing six frames, took approximately 1 min. As such, automatic tracking provides measurements across the entire video in a much shorter time.

The automatic measurements were less reproducible than manual measurements in contrast to a previous study (Miyoshi et al., 2009). Automatic measurements were observed to be affected by the initial fascicle that was identified in the first frame. At times, the selected fascicle would move over a vein or another stationary structure, or the fascicle would appear to move out of the plane of the ultrasound. This would cause the tracked fascicle to “lag” and at times track the fascicle on either side of the initial fascicle. During unsupervised tracking, the first tracking attempt was accepted regardless of tracking quality, which we believe contributed to the lower agreement between manual and automatic tracking (CMC > 0.83). Supervision removes this issue because it allows the user to simply identify a different fascicle that does not have this issue (Fig. 1). The efficacy of this is demonstrated by the very strong agreement between manual and automatic measurements (CMC > 0.94) and low RMES for fascicle length (RMSE = 3.31 ± 1.53 mm) and pennation angle RMSE = 4.12° ± 1.98°). The improvement of tracking due to supervision is supported by the work of Aeles et al. (2017) where they observed that providing examiners with the ability to watch the video prior to fascicle identification improved the reliability of fascicle identification.

Similar to other studies, we found that automatic measurements of pennation angle were less reproducible than fascicle length (Zhou, Chan & Zheng, 2015) and also had higher RMSE errors. At least for the present study, we believe that this increased error in pennation is due to trigonometry. When the fascicle is contracted, small variations in fascicle length results in large variations in pennation angle (Figs. 1E and 1F). It is apparent that tracking is poor and the measurements of fascicle length (57.8 mm) and pennation angle (32°) are incorrect (Fig. 1E). Reinitializing the program and identifying a different fascicle enables better tracking (Fig. 1F). The measurements of fascicle length (50.6 mm) and pennation angle (49°) are significantly more accurate. The incorrect track results in a 12.5% over-estimation of fascicle length and 53.1% under-estimation of pennation angle relative to the well-tracked video. Aeles et al demonstrated that tracking a fascicle backward in time from contracted to relaxed state did not improve the accuracy of the fascicle length measurement relative to traditional forward tracking. This supports our results that fascicle length measurements did not degrade as the video progressed (Table 5). We observed that error in pennation angle measurements did increase over time, however. Therefore, we suggest that future studies investigating the role of tracking direction on pennation accuracy are warranted.

Automatic tracking performance was dependent on ultrasound acquisition rate and quality. During pilot testing, we acquired images at nearly half the frame-rate (30 frames/s) of our reported data (60 frames/s). We found that tracking was poor, possibly due to the large displacement of tracked points in fast contraction velocities. Based on these findings, we suggest that ultrasound images should be acquired as quickly as possible while still maintaining good image quality. Recent advances in high frame rate ultrasonography have increased the possible capture rates to 500–2,000 frames/s to study very fast ankle rotations (Hauraix et al., 2015), although the trade-off between image quality and frame rate is based on specific hardware specifications. Additionally, we found that inter-subject muscle variability affected tracking quality. Most notably, subjects with clearly identifiable fascicles in the first frame that remained visible throughout the entire contraction tracked better than trials that became less clear later in the contraction (Fig. 1). Tracking quality was partly dependent on defining the fascicle in the first frame. A potential improvement to this approach would be the development of a process to automatically identify the fascicle in the first frame to reduce variability. One possible approach demonstrated by Zhou, Chan & Zheng (2015) used images transforms to automatically identify line-like structures of the fascicle. This approach could be used to automatically establish a ROI around each of these structures in the first image.

This study was affected by several limitations. Similar to other automatic tracking approaches, we approximated muscle fascicles and aponeuroses as straight lines, which may lead to measurement errors where fascicle length is under-approximated due to fascicle curvature (Cronin et al., 2011; Gillett, Barrett & Lichtwark, 2013; Zhou, Chan & Zheng, 2015; Farris & Lichtwark, 2016). In our data, we did not observe very much fascicle curvature, even in fully contracted state. Fascicles in the gastrocnemius do not curve as much as fascicles in other muscles such as the vastus medialis (Gillett, Barrett & Lichtwark, 2013) or the biceps femoris (Seymore et al., 2017; Aeles et al., 2017). If significant amount of fascicle curvature is observed, we suggest that users exercise judgment as to whether this straight-line approximation is valid. We should note that two out of the three examiners who analyzed the ultrasound images were relative novices. While these examiners were trained by an experienced examiner, novices have been shown to be less reliable than expert and automatic tracking approaches (Miyoshi et al., 2009). However, our findings show that even novice examiners have very strong reproducibility when manually measuring fascicle length (ICC > 0.93) and pennation angle (ICC = 0.9).

Another limitation is that we did not manually digitize all of the frames across the entire contraction. Each trial contained between 50 and 150 frames which was effectively down sampled to six frames for manual measurements. Our rationale for the limited number of manual measurements was to prioritize the number of subjects (*n* = 5), rotational velocities (*n* = 5), number of trials (*n* = 3), and number of repeated measurements (*n* = 3) that each examiner (*n* = 3) analyzed. While other validation studies have had a larger number of manual measurements for each ultrasound video, these studies had a limited number of subjects (Miyoshi et al., 2009), a limited number of conditions (Zhou, Chan & Zheng, 2015), or digitized only a subset of the ultrasound videos (Cronin et al., 2011; Gillett, Barrett & Lichtwark, 2013). In addition, the six manual measurements were made at the same approximate time points across all contractions. As such, we were unable to closely identify in which time windows of a contraction errors occurred. We did observe that RMSE decreased over time for fascicle length measurements and increased for pennation angle measurements. Another limitation of this study is that we used an iterative tracking approach where errors in earlier frames can accumulate over the course of the track. We demonstrated that supervision of tracking increased the accuracy of the track, especially in later frames (Table 5). To reduce the accumulation of errors for longer videos in which multiple cycles of contractions are tracked, Ultratrack has a powerful set of tools to account for the propagation of errors such as keyframe adjustments and the ability to make manual corrections (Farris & Lichtwark, 2016). As our program is a command window initiated tracking program rather than a full suite of tools wrapped in a graphical user interface, we hope that our tracking paradigm can be incorporated into a tool with similar features in the future.

## Conclusions

Accurately quantifying muscle architecture from ultrasound imaging during maximal effort tasks provides researchers and clinicians with an important tool for understanding the structure-function relationship that underpin muscular injury, disease, and recovery. This study introduced a novel fascicle and aponeuroses tracking approach that quantifies architectural parameters of individual muscle fascicles using a commercially available point tracker. We demonstrated that our proposed automatic fascicle tracking algorithm is repeatable and reproducible across a wide range of angular velocities even when used by different examiners across different days. It showed strong agreement with manual measurements, especially when used in a supervised manner. When initialized from the first frame with identical starting positions to Ultratrack, our program exhibited less error while also providing accurate measurements of pennation angle.

## Additional Information and Declarations

### Data Availability

The following information was supplied regarding data availability: The complete data is available at: https://github.com/joshrbaxter/ultrasound_tracking (code) and John F Drazan, Todd J Hullfish, & Josh R Baxter. (2019). An automatic fascicle tracking algorithm quantifying gastrocnemius architecture during maximal effort contractions (Version ver1) [Data set]. Zenodo. http://doi.org/10.5281/zenodo.2598553. The data set includes the tracking code (MATLAB), ultrasound videos (in proprietary TVD format as well as MP4 and AVI), motion capture data (ultrasound trigger), and isokinetic dynamometer data (Biodex). We have also provided an example folder that allows for turn-key testing of the tracking algorithm. Additionally, the tracked data and videos are also included in this repository.
